# Changes in harm reduction service providers professional quality of life during dual public health emergencies in Canada

**DOI:** 10.1186/s12954-024-00966-3

**Published:** 2024-02-22

**Authors:** Sheena Taha, Samantha King, Sara Atif, Eliza Bate

**Affiliations:** Canadian Centre on Substance Use and Addiction, 75 Albert St, Ottawa, ON K1P 5E7 Canada

**Keywords:** Professional quality of life, Burnout, Secondary traumatic stress, Job satisfaction, Vulnerability to grief, Drug toxicity emergency, Covid-19

## Abstract

**Background:**

Harm reduction (HR) is a critical response to the pronounced toxicity deaths being experienced in Canada. HR providers report many benefits of their jobs, but also encounter chronic stress from structural inequities and exposure to trauma and death. This research study sought to quantify the emotional toll the toxicity emergency placed on HR providers (Cycle One; 2019). Study objectives were later expanded to determine the impact of the ongoing toxicity as well as the pandemic’s impact on well-being (Cycle Two; 2021).

**Methods:**

Standardized measures of job satisfaction, burnout, secondary traumatic stress, and vulnerability to grief were used in an online national survey. Open-ended questions addressed resources and supports. HR partners across Canada validated the findings and contributed to alternative interpretations and implications.

**Results:**

651 respondents in Cycle One and 1,360 in Cycle Two reported moderately high levels of job satisfaction; they reported finding great meaning in their work. Yet, mean levels of burnout and secondary traumatic stress were moderate, with the latter significantly increasing in Cycle Two. Reported vulnerability to grief was moderate but increased significantly during COVID. When available, supports lacked the quality necessary to respond to the complexities of HR workers’ experiences, or an insufficient number of sessions were covered through benefits. Respondents shared that their professional quality of life was affected more by policy failures and gaps in the healthcare system than it was by the demands of their jobs.

**Conclusion:**

Both the benefits and the strain of providing harm reduction services cannot be underestimated. For HR providers, these impacts are compounded by the drug toxicity emergency, making the service gaps experienced by them all the more critical to address. Implications highlight the need for integration of HR into the healthcare system, sustainable and reliable funding, sufficient counselling supports, and equitable staffing models. Support for this essential workforce is critical to ensuring the well-being of themselves, the individuals they serve, and the health of the broader healthcare system.

## Introduction

Over the last 20 years, drug-related harms — particularly those related to opioids and opioid analogues — have increased markedly, leading to a national public health emergency in Canada [1]. Between January 2016 and March 2023, 22,828 people died because of opioid toxicity and 38,514 people were hospitalized for opioid- and stimulant-related poisoning [2]. While staggering, these numbers do not capture the full range of individuals, families, friends, and communities that are grieving, and continuing to be impacted by substance-related harms.

With fentanyl appearing in 55% of toxicity deaths in Canada in 2019, 80% in 2020 and 87% in 2021 [2], harm reduction (HR) has become a critical response to the increasingly toxic drug supply. Evidence related to the effectiveness of HR services has been well established [3–7], yet many HR services are provided by unregulated workers, volunteers, or both to fill gaps in healthcare services [8]. These individuals often have their own lived or living experience (LLE) with substance use [9]. Providers with LLE report many benefits of their jobs, including purpose and meaning in their daily work and being valued for their expertise. However, they also report challenges to their roles, including discrimination, job instability and a lack of benefits or compensation [8–12].

Many HR services in Canada are under resourced and unsupported [9]. Providers experience chronic, daily stress from structural factors that create a precarious and inequitable working environment such as a lack of resources to fund and support their work environments [1, 11, 13, 14], instability in long-term vision for their work (e.g., short-term approvals for overdose prevention sites [15, 16]; a lack of recognition of the expertise that individuals with lived or living experience have [1, 8, 17]; and stigma toward substance use in general and harm reduction services specifically [1, 6, 17]. Furthermore, the nature of HR work can be emotionally taxing with constant exposure to trauma and death. Many individuals providing harm reduction services have lived experiences and personal ties with those who have died from drug toxicity. As a result, many HR providers live their daily lives while burdened with grief and fear of further loss among their friends, family, and community [1, 11, 14, 18, 19]. A potential outcome of repeatedly witnessing these harms is the development of burnout, compassion fatigue and secondary traumatic stress [11, 20].

In the midst of the toxicity emergency in Canada, the World Health Organization declared COVID-19 a global pandemic in March 2020 [21]. Individuals who use substances were at increased risk of harms from COVID-19 because they had a greater likelihood of underlying health conditions and of living in settings where social distancing and isolation were not possible. They were also at increased risk for substance-related harms, such as experiencing a toxicity event while using a substance alone or experiencing withdrawal while isolated [22].

Thus, the many challenges to providing HR services were worsened by the COVID-19 pandemic. Many programs had to stop or reduce services when governments initiated COVID restrictions, including lockdowns [23]. Additionally, both people who use drugs and care providers said physical distancing and virtual-only contact were barriers to treatment services as these changes dehumanized social connections and lessened opportunities to build trust [24]. And yet, clients reported that the continuation of HR services were lifelines, providing safety and stability during major interruptions in other services [24].

The well-being of frontline healthcare providers declined during previous world-wide pandemics with adverse psychological effects, such as stress and anxiety most often observed [25]. 64% of physicians reported occupational stress (which leads to emotional exhaustion) during COVID, as compared to reports ranging from 24 to 46% pre-COVID [26]. A survey of providers in Canada who continued to provide harm reduction and supportive housing services during COVID-19 revealed that 80% of respondents reported a decline in their mental health [27]. While the initial objective of this study was to gain a national understanding of how the drug toxicity emergency was affecting individuals providing HR services, we later expanded the objectives to identify how both the pandemic and the ongoing drug toxicity emergency were affecting these individuals.

## Method

This study applied an integrated knowledge mobilization approach ensuring that HR providers from across Canada were involved throughout the project. Harm reduction organizations from each jurisdiction across Canada were invited to have one or more representatives participate as partner advisors the study (*n* = 8). Where provincial or territorial organizations did not exist, community services were engaged from major cities in the jurisdiction (*n* = 7). Partners also provided recommendations for other harm reduction focused organizations that would have a vested interest in the topic (i.e., Harm Reduction Nurses Association, Centre for Innovation in Peer Support) and these organizations were invited to participate as well (*n* = 2). Partners came from multiple backgrounds including nursing, public policy, social outreach, lived experience, etc., and all were currently focused on providing harm reduction services and supporting individuals on the front lines. Representatives of jurisdictional and community harm reduction services participated in multiple consultations. During the first set of consultations (two virtual meetings) partners validated the need for this research, identified key themes to be explored, refine research questions and came to consensus on the appropriate use of language for the project. A second round of consultations were held at the conclusion of CYCLE ONE and CYCLE TWO (one meeting for each cycle, and one-on-one consultations as necessary due to schedule conflicts). In these sessions findings from quantitative and qualitative analyses were shared. Partners validated or provided alternative interpretations of the results, identified on the ground implications of the findings, and suggested actions to be implemented. Finally, partners reviewed the final draft of this paper and provided any final feedback or considerations. This partner involvement was key to ensuring that the conclusions were appropriate and fit with the experiences and needs of service providers. We appreciate the participation of these partners whose contributions greatly enriched the document (see Acknowledgements).

To facilitate timely data collection and to reach respondents across the country, both surveys were administered online. All materials were available in English and French. The Advarra Institutional Review Board provided ethics approval for both cycles of the research performed for this report.

### Measures

#### Professional quality of life

The 30-item Professional Quality of Life (ProQOL) scale was used to assess positive and negative experiences of providing HR services to individuals who use drugs. Subscales assessed levels of compassion satisfaction and compassion fatigue, including burnout and secondary trauma, in the last 30 days [28]. The ProQOL does not offer an option to generate a meaningful composite score, therefore the sum results from each subscale are reported separately and could range from 10 to 50. Previous studies have evaluated the psychometric properties of the scale [29] and in a sample population similar to ours, it has demonstrated satisfactory consistency and validity [30].

The compassion satisfaction subscale of the ProQOL scale measured the “pleasure derived from being able to do your work well” [31]. This may include feeling good about helping others through your work and feeling positivity toward your colleagues and your ability to contribute to the work setting. A higher score on this subscale reflects that a person derives great professional satisfaction from their work [28, 31].

The compassion fatigue subscale measured the negative consequences of helping others [29]. This is a work-related phenomenon many refer to as a cost of caring and it contributes to a reduction in compassion in health care [32]. In the context of the ProQOL measure, compassion fatigue is measured using two subscales: burnout and secondary traumatic stress.

Burnout is associated with feelings of hopelessness and difficulties in dealing with work or in doing a job effectively, whereas econdary traumatic stress can result from hearing stories about traumatic things that have happened to others [31].

The ProQOL has been used with medical health professionals, social service employees, humanitarian workers [28], hospice and palliative care professionals [33–35], and chaplains [34]. Normative benchmarks among professional caregivers working with survivors of trauma have been established [36] and are reported in Table 1.

**Table 1:** Average professional QoL subscales scores in various studies, M (SD)

Subscale	Cycle 1	Cycle 2	Caregivers interacting with trauma survivors (De La Rosa et al., 2018)	Emergency department nurses (Hunsaker et al., 2015)	Hospital and primary healthcare nurses (Ruiz-Fernández et al., 2020)	Healthcare workers during COVID (Buselli et al., 2020)
Compassion satisfaction	36.7 (6.1)	35.5 (7.0)	37.7 (6.5)	39.7 (6.3)	35.48 (7.4)	38.2 (7.0)
Burnout	26.0 (4.7)	26.6 (5.5)	22.8 (5.4)	23.66 (5.9)	23.44 (5.3)	19.8 (5.0)
Secondary traumatic stress	25.6 (7.4)	28.7 (6.7)	16.7 (5.7)	21.57 (5.4)	20.74 (7.8)	18.0 (5.6)

#### Adult attitude to grief

The nine-item Adult Attitude to Grief scale assessed vulnerability to grief from losing people because of the drug toxicity emergency [37]. Questions include three subscales: resilient, controlled, and overwhelmed. The sum of the scores across all subscales represents the respondent’s overall vulnerability to grief and could range from 0 to 36 [38]. The scale was found to be psychometrically promising for identifying vulnerability to grief, with acceptable consistency and validity [37].

Higher vulnerability to grief contributes to difficulties in managing loss and its consequences emotionally, socially, and practically. It is considered the opposite end of the spectrum from resilience in the face of loss [38]. This measure has been used among clients from community- or hospital-based bereavement services [38].

### Data collection

#### Cycle one

Data was collected online from July 30 to Sept. 30, 2019, during the ongoing drug toxicity emergency. Respondents provided demographic data and completed standardized measures of Professional Quality of Life and vulnerability to grief. Open-ended questions were included to allow participants to add contextual information and to comment on resources and supports that were available or could be beneficial if implemented. For example, “Is there anything about your professional quality of life that you’d like to share?” and “Please describe what supports and resources you feel are in place that are helpful to you in your role?”.

#### Cycle two

With the COVID-19 pandemic being declared less than six months after Cycle One was completed, we wanted to determine the effect of the pandemic on the same population. A revised survey was used in the second cycle of this research project to collect data from January 27 to March 8, 2021. This cycle included the measures used in Cycle One, with additional open-ended questions about changes in the work environment and to workers well-being as a result of the pandemic. For example. “Have your feelings about work or the ways in which you work impacts your quality of life changed since the onset of the COVID-19 pandemic” and “Have your grief responses and feelings changed since the onset of the COVID-19 pandemic?”.

### Participants and recruitment

Eligible participants were people living in Canada who self-identified as HR providers and were the age of majority (considered a legal adult) in their jurisdiction. Partners involved in the study’s development disseminated the survey to their networks of HR providers, through social media, e-mail, list servs, and organizational newsletters to recruit using snowball sampling.

In Cycle One, individual responses were anonymous, however, if participants shared their email, they were eligible to enter a draw with a one in five chance to win a $20 coffee shop e-gift card. In Cycle Two, compensation was revised based on partner feedback and all participants who completed a valid survey and provided an email address were offered a $20 gift card to their choice of an online retailer, a grocery store or coffee shop.

### Data analysis

The quantitative survey data were analyzed using the Statistical Package for the Social Sciences version 22. Survey respondents who included only demographic information or endorsed the same response (e.g., only selecting “Very often”) for each question within any of the three scales were excluded from the analyses. Sample sizes differed for each of the variables reported as respondents filled out the survey to varying degrees. Analysis of covariance (ANCOVA) was used to determine if any outcome variables of interest (compassion satisfaction, burnout, secondary traumatic stress) differed between the two cycles. Gender, age, and number of years working in HR were included as covariates for these analyses.

Qualitative analysis on the open-ended questions was conducted through the creation of code lists. The coding allowed for the identification of overall themes and for a summary response to be developed for each individual question. To create the code lists, first, a preliminary review of a subset of the survey responses (50–60%) was manually conducted be a team of coders to get a sense of the range and variety of survey answers. Second, a comprehensive code list was developed based on the entire set of responses, where each code represented a theme. Finally, each individual response was manually assigned between one and five codes based on its content.

## Results

### Demographics

**Fig. 1:**
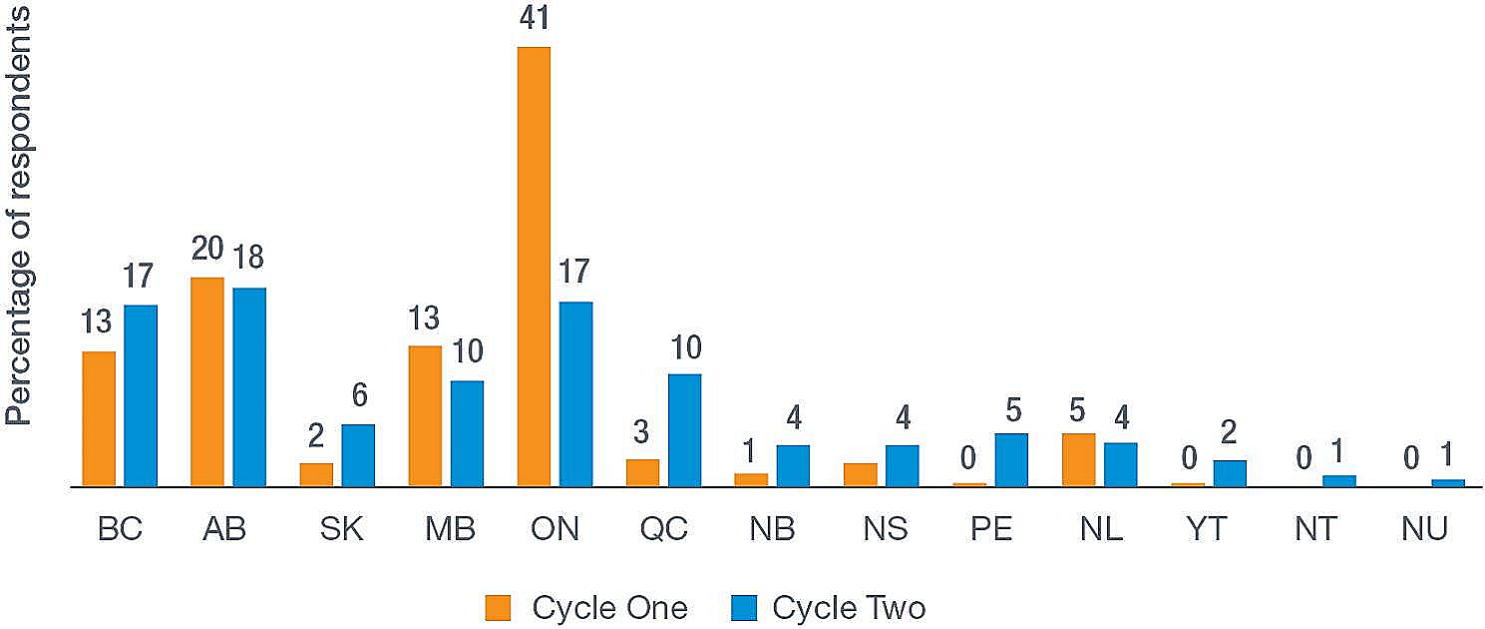
Provincial and territorial distribution of respondents. **Abbreviations**: British Columbia (BC), Alberta (AB), Saskatchewan (SK), Manitoba (MB), Ontario (ON), Quebec (QC), New Brunswick (NB), Nova Scotia (NS), Prince Edward Island (PE), Newfoundland and Labrador (NL), Yukon Territory (YT), Northwest Territories (NT), Nunavut (NU)

Six hundred, fifty-one valid surveys were completed in 2019, and 1,360 in 2021. The regional distribution of respondents for each survey cycle is presented in Fig. 1 with most respondents located in Ontario, Alberta, British Columbia, and Manitoba. In 2019, most respondents identified as women, whereas in 2021, most identified as men (see ‘Gender’, Table 2). Respondents in both cycles tended to be between 25 and 44 years of age (see ‘Age’,Table 2). Level of education varied among participants, with a large proportion completing some form of postsecondary education in both cycles (see ‘Highest level of education’, Table 2). Most respondents lived in urban or suburban environments, reported working full time and had been working in the HR sector for five years or less (see ‘Size of community’, “Employment status’, and ‘Time working in harm reduction’, Table 2).

**Table 2:** Demographic Characteristics of Survey Participants in Each Cycle, *n* (%)

Characteristic	Cycle One, 2019 (*n = 651)*	Cycle Two, 2021 (*n =* 1,360)
**Gender**		
Women	501 (77.3)	604 (46.7)
Men	130 (20.1)	678 (52.4)
Gender diverse	17 (2.6)	11 (0.9)
**Age**		
18 to 24 years old	39 (6.2)	214 (18.7)
25 to 44 years old	410 (64.8)	828 (72.2)
45 to 64 years old	169 (26.7)	101 (8.8)
65 years or older	15 (2.4)	4 (0.3)
**Highest level of education completed**		
Less than high school	8 (1.2)	94 (6.9)
High school diploma	17 (2.6)	181 (13.3)
General Ed. Dev. (GED) or Adult Basic Ed. (ABED)	3 (0.5)	88 (6.5)
Some college or technical school	28 (4.3)	180 (13.2)
College or technical school graduate	125 (19.3)	310 (22.8)
Undergraduate university degree	285 (43.7)	356 (26.2)
Professional degree (e.g., law, medicine)	64 (9.9)	78 (5.7)
Graduate degree (master’s or doctorate)	115 (17.8)	48 (3.5)
Other	2 (0.3)	16 (1.2)
**Size of community**		
Urban or suburban (in a city or town)	572 (87.9)	905 (66.5)
Rural (short drive to city or town)	55 (8.4)	305 (22.5)
Remote or isolated (great distance from city/town)	23 (3.5)	191 (14.0)
Not sure	—	10 (0.7)
Prefer not to say	—	4 (0.3)
**Employment status**		
Full-time employee	491 (75.4)	735 (54.0)
Part-time employee	127 (19.5)	380 (27.9)
Full-time volunteer	5 (0.8)	103 (7.6)
Part-time volunteer	36 (5.5)	212 (15.6)
**Time working in harm reduction**		
2 years or less working in harm reduction	190 (29.2)	380 (27.9)
3 to 5 years working in harm reduction	147 (24.7)	393 (28.9)
6 to 10 years working in harm reduction	133 (22.4)	211 (15.5)
11 to 20 years working in harm reduction	102 (17.2)	62 (4.6)
21 years or more working in harm reduction	22 (3.7)	13 (1.0)

*Note.* — = not available

### Professional QoL

**Fig. 2:**
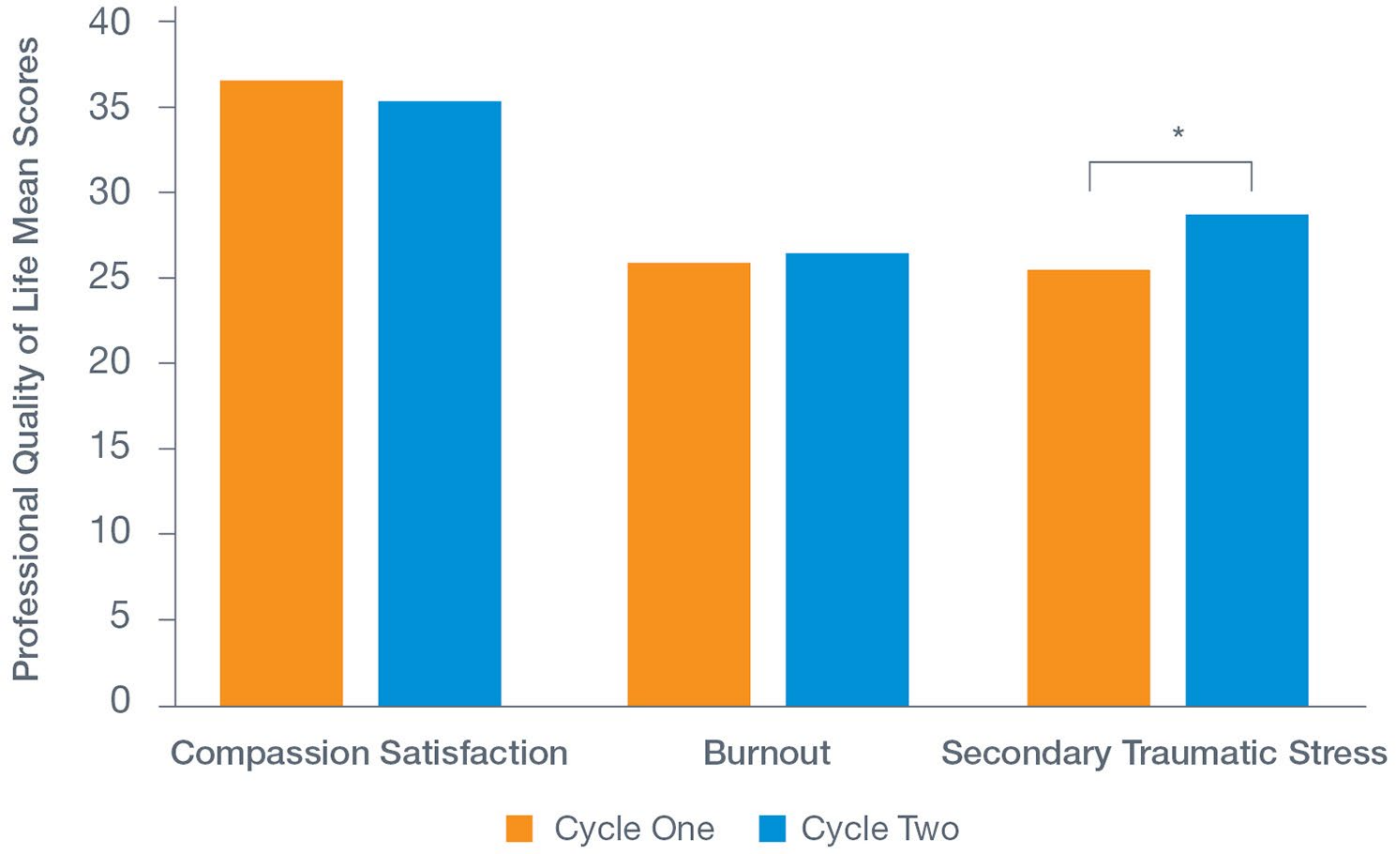
Professional quality of life scores by cycle

We utilized the ProQoL to assess the positive and negative experiences of HR service provision. Reported experiences of compassion (job) satisfaction were moderately high and did not differ between Cycle One (*M* = 36.7, *SD* = 6.11) and Cycle Two (*M* = 35.5, *SD* = 7.00; *F*[1, 1491] *=* 0.325, *p* = .569; Fig. 2).

Levels of burnout were moderate and remained consistent from Cycle One.

(*M* = 26.0, *SD* = 4.7) to Cycle Two (*M =* 26.6, *SD =* 5.5; *F*[1, 1491] = 3.73, *p* = .054) (Fig. 2).

Levels of secondary traumatic stress were initially moderate and increased significantly from

Cycle One (*M =* 25.6, *SD* = 7.4) to Cycle Two, (*M =* 28.7, *SD* = 6.7; *F*[1, 1491] = 16.25, *p* < .001) (Fig. 2).

To allow respondents to provide more details about their professional quality of life, we asked “Is there anything else about your professional quality of life that you’d like to share. In total, 38% (*n* = 250) of CYCLE ONE respondents and 18% (*n* = 242) of CYCLE TWO respondents provided a response to this question. Overall, themes revealed that respondents enjoyed and believed in the importance of their work,, while at the same time outlining the need for additional services and supports for clients and for the sector overall.

*I love my work and the people I am privileged to serve, although not having enough community-based services to help them is extremely difficult. (CYCLE ONE)*

*I love my job and simultaneously wish it didn’t have to exist. I couldn’t imagine doing nothing about the drug toxicity crisis. It is an honour to be paid for something I am so passionate about, but also hard to be engaged in what seems like such an endless battle with governments to value the lives of people like me. (CYCLE TWO)*

Respondents also described frustration and dissatisfaction with their professional quality of life such as wanting to see improvements to working conditions, increased benefits and compensation, improved services and supports for clients, increased government support and funding, increased awareness of and support specific for the drug toxicity emergency and HR work, and improved management.

*The system is what affects the quality of my professional life, rarely the clients. The system calls it burnout — I prefer the term constant moral assault. (CYCLE ONE)*

*Agency systems, governments, policies, and politics make it hard to provide the best care for people in the community that need HR services. (CYCLE ONE)*

CYCLE TWO respondents reported COVID-19 pandemic-related issues impacting their professional quality of life, such as reduced services and public health restrictions due to the pandemic.

*During the pandemic, I have lost access to things that improve my mental health, like flexible scheduling and vacation time. Demand for the services my team provides has never been higher, and there has been no acknowledgement of the risks that we are taking or the services we are providing. (CYCLE TWO)*

*COVID-19 has increased stress levels when interacting with people because of the restrictions and the fear of infection. (CYCLE TWO)*

### Work-related changes during the COVID-19 pandemic

CYCLE TWO respondents were asked whether their feelings about their work or the ways in which their work impacts their QoL changed since the start of the COVID-19 pandemic. Almost a quarter of respondents (24%, *n* = 326) confirmed that they had. To better understand work-related changes, respondents were asked to describe the changes and their interpretation of the reasons for changes. The main theme reported by 39% (*n* = 128) respondents, was the impact on the type and quality of care they could provide clients (including lack of supplies, inability to assist clients who lacked access to internet or other communication devices

*Many barriers have arisen for users of safe consumption sites: masks, social distancing, protocols for access for some facilities, etc. A lot of the population struggling with addiction get frustrated with the rules in place, so they choose to just use alone. Some die because of this.*

*More barriers to services for client’s which makes my job harder, and I feel more helpless to empower people.*

Other themes included reduced program and service accessibility and availability for clients, increased workload, increased fatigue and burnout, increased isolation, limited socialization, and changed mental health status.

*The workload keeps piling up. Personally, I feel more tired than before.*

*I’m aware that my own personal stress leaves me with less to give or to offer at work. I have to be very measured with my energy and conserve it where I can. I can’t extend myself as much as I did pre-COVID.*

A few respondents mentioned an increase in positive work-related changes since the start of the pandemic which included the ability to help clients, the community and society in general, as well as finding their work more meaningful and motivating.

*I feel that the work has become more vital. With COVID-related shutdowns, moving or changes of services, it has become more important to be a reliable, consistent source of HR resources including supplies.*

### Vulnerability to grief

Generally, levels of vulnerability to grief were moderate in CYCLE ONE (*M* = 14.91, *SD* = 4.14) and increased significantly in CYCLE TWO (*M* 17.16, *SD* = 3.74) *F*(1,1466) = 42.54, *p* < .001.

We asked respondents whether there was anything else they wanted to share about their grief: 22% (*n* = 142) of CYCLE ONE respondents and 15% (*n* = 200) of CYCLE TWO respondents shared additional information. Of these, many indicated that although support through the grieving process was important, it was not always available or accessible, and that it is difficult to deal with grief in general.

*I find it is always a struggle between wanting to support those affected by the death in the moment and being gentle with yourself as you are also grieving. It’s a difficult balance. (CYCLE TWO)*

Another common theme emerging from respondents was the importance of acknowledging that everyone deals with grief differently, and that grief can be difficult to process when you work with individuals who experience grief and loss regularly.

*When a patient [or] client dies, it is actually necessary for me to take a short mindful break and then continue my workday. It’s difficult to explain why exactly I feel the need to keep going. I think I do this for a variety of reasons. For example, I believe I need to keep working, otherwise the loss of one could contribute to the loss of another, and I won’t allow that to happen. I also process loss and death over a period of time, perhaps several days, and can continue to process the person’s death while I am continuing to live. (CYCLE ONE)*

*Grief for me is always going to weigh heavy. I find one of the hardest aspects of grief is finding a balance between opening up to new life and new joy while still engaging with the grief as such an important part of present experience. It is a confusing thing to have to accomplish, and in my experience, it has been difficult to find valuable guidance in this area as well as the opportunity to express and share experience with grief. (CYCLE ONE)*

CYCLE TWO respondents described experiencing more death and grief because of the COVID-19 pandemic as well as changes in their mental health and well-being.

*We lose someone daily. I feel like we don’t got time to grieve. (CYCLE TWO)*

*Two coworkers in our tiny organization have passed away since the beginning of the pandemic. This is another layer on top of the frequent deaths of clients. (CYCLE TWO)*

### Changes in vulnerability to grief during COVID-19

Nearly 10% (*n* = 130) of all CYCLE TWO respondents reported changes in their grief responses since the start of the pandemic. The main theme emerging from respondents was increased amounts of death and grief.

*I think the pandemic has made me acutely aware of the increased risks for those who use substances and therefore the increased risk of drug toxicity death. (CYCLE TWO)*

Additional challenges created by the pandemic included: following public health guidelines, social isolation, inability to gather with family after loss) and unhealthy changes in mental health and well-being (e.g., increased fear, anxiety, feelings of sadness).

*I think I’m more sensitive. Due to the length of the pandemic, some feelings of frustration and helplessness. Need to be aware and address these feelings and correcting myself (CYCLE TWO)*.

*We are more isolated, so it makes it hard to receive support for grieving. (CYCLE TWO)*

Some respondents described adopting a more positive outlook, including being more optimistic, cherishing life and being better equipped to cope with grief and loss.

*The pandemic has given me new skills to cope, and the experience of this loss has shown me some new skills that I have for coping. (CYCLE TWO)*

### Resources, programs and supports: findings

To determine what workplace resources were available, we asked respondents to tell us what supports were already in place that are helpful to them in their role. 76% (*n* = 494) of CYCLE ONE respondents and 44% (*n* = 599) of CYCLE TWO respondents provided an answer. Among those, many indicated having access to the services they needed, which included employee-specific supports (e.g., counselling, team meetings and debriefings, benefits), professional and agency supports (e.g., staffing support like nurses and outreach teams) and supportive supervisors. Several CYCLE TWO respondents included availability of COVID-19 specific resources such as pandemic-related supports, equipment and supplies, and volunteer resources.

*Supportive team who meets regularly to support one another and discuss difficult cases. The availability of [an] employee assistance plan to provide paid counselling service if necessary. (CYCLE ONE)*

*The consistent support of my peers, coworkers and like-minded individuals who share the same goal. Having places like [name of local service] also are helpful for us staff in the community, and it’s helpful to redirect our clients to them as well as for clean supplies [or] resources. (CYCLE TWO)*

*We have a professional [employee assistance] program, which gives us access to counselling [and] therapy. My team manager is quite supportive. My team is very supportive. (CYCLE TWO)*

### Lack of supports for work and well-being in the workplace

Respondents were also asked what they felt was lacking in terms of support for their work and well-being. 69% of CYCLE ONE respondents (*n* = 449) and 34% of CYCLE TWO respondents (*n* = 457) provided an answer to this question. Common themes included the need for increased employee-specific supports and resources (e.g., better communication, increased benefits, more debriefings, and team meetings), resource supports (e.g., counselling, mental health supports), educational supports (e.g., training) and professional and agency supports (e.g., hire more staff to keep up with demands),

*Lots of nice talk about our values but not backed up with action; lack of resources to do the work; lack of education and a commitment to HR principles across the organization; trauma therapy easily accessible and as long as it needs to be. (CYCLE ONE)*

*My insurance does not cover enough counselling. $500 a year is not enough. (CYCLE TWO)*

*In [jurisdiction name] with the merger into a provincial system, local supports have been eliminated and budget lines for any extras have disappeared. We are told funding is in a crisis and there’s no money for anything. Job and budget cuts are pending. There is plenty of opportunity for change; however, leadership would need to see value in employee engagement, employee training, employee growth and employee retention throughout the continuum of addiction services. (CYCLE ONE)*

*More training for all levels of workers, safe spaces for community members to access while using. (CYCLE TWO)*

CYCLE TWO respondents also reported the need for supports related to COVID-19, such as social distancing, personal protective equipment as well as supplies and physical safety supports for clients (e.g., housing supports, emergency shelter spaces).

*Increase protection measures, take temperature checks on personnel and distribute masks. (CYCLE TWO)*

*Need more funding for increase[d] hours and staff. Staff should be better supported to isolate if [they] have symptoms and return to work if negative… not punished for sick time because they stayed home when experiencing symptom[s]. (CYCLE TWO)*

*Residential treatment programs are unavailable. There is a severe lack of housing for vulnerable populations to stabilize them and support them to get to treatment. In-patient services are working poorly with community services. There is no collaboration. (CYCLE TWO)*

### COVID-19 pandemic-related changes to resources and supports

CYCLE TWO respondents were asked whether the types of resources, programs, and supports they had access to or how they accessed these services had changed since the start of the COVID-19 pandemic. In total, about 20% (*n* = 279) answered “Yes.”

We asked respondents to describe what had changed and the reasons what they attributed it to. Overall, lack of services and resources (e.g., limited services, discontinued services) and increased virtual services were mentioned most often and reported by 43% of respondents to that question.

*Very difficult for clients to access health care during a pandemic. However, virtual OAT [opioid agonist therapy] programs have been a real positive for clients required to isolate during COVID. (CYCLE TWO)*

*There doesn’t seem to be as much room in shelters for our clients or warm places in general. (CYCLE TWO)*

Other themes reported because of the pandemic included inability to access services (e.g., reduced hours, limited capacity for clients, longer wait times) and lack of in-person services (e.g., human experience, personal connection).

*Lack of in-person services, which makes it harder to build therapeutic relationships. (CYCLE TWO)*

*People are required to answer screening questions, which may trigger people with anxiety or chronic illnesses [and I] always feel terrible. Face-to-face is not allowed and misunderstandings are increased as to the needs to be met for some people. Some programs and resources are help for COVID-related topics only. (CYCLE TWO)*

Most respondents described struggles they were facing, however, some discussed positive outcomes, such as increased program availability, improved communication and additional COVID funding.

*The silver lining of the pandemic is that some of the funding resources we were needing are now available to us to help deal with the pandemic. However, the bad side of this is that strict limitations on services have pared down our already inadequate mental health and addictions programming. … But we can buy all the masks and bleach we could ever need (if they’re available). (CYCLE TWO)*

The Importance of Harm Reduction Work and Support for Employees The final question of both surveys asked if there was anything else individuals wanted to share about their work and their experiences. About 28% (*n* = 183) of CYCLE ONE respondents and 11% (*n* = 146) of CYCLE TWO respondents answered this question. The major themes included liking what they do and knowing HR work is important.

*I simply love my work … I don’t even think of it as work most days … I think of it as my duty to my fellow person. The worst part is all the paperwork, the people aren’t the work. It is a joy when I can find something that can alleviate some of that hardship and suffering. The frustration in this work comes from the ignorance of others and their treatment of PWUS [people who use substances]. People are dying because they avoid healthcare and other services. (CYCLE ONE)*

*I have the best workplaces and coworkers who really get what HR is all about! (CYCLE TWO)*

Respondents also indicated the need for employee-specific services and supports, HR -specific resources, more government funding, and increased efforts to reduce stigma about HR work, homelessness, and addiction.

*More training on how to provide HR in a supportive manner. (CYCLE ONE)*

*I think overall it is not my specific workplace or my specific self-care practices that would support me best to do this work — it is systemic change in the way that we treat people living in poverty, racialized people, people with disabilities, women and trans people, and people who use drugs. If the system were better designed, I think a lot of the fatigue that people experience in HR -based jobs could be mitigated. (CYCLE ONE)*

*Being a HR worker right now is extremely emotionally demanding. We are losing a lot of people, and it feels like the rest of the world doesn’t care or acknowledge this. It’s like a war zone. (CYCLE TWO)*

## Discussion

The initial purpose of our study was to provide a national picture of the experiences of frontline HR providers in Canada. With the emergence of the COVID-19 pandemic, a second cycle of data was collected to examine the effects of this additional challenge, and to identify the compounding effects of the ongoing drug toxicity emergency. Both surveys were enhanced by the participation of representatives from harm reduction organizations across Canada. They validated themes and rationale, helped interpret the results and endorsed the implications of the findings. The input of these individuals is captured in the discussion below where consultation partners are referenced.

Service providers experienced moderately high levels of compassion satisfaction from their work across survey cycles. Levels of burnout and secondary traumatic stress were concerning, higher than those established as benchmarks among professional caregivers who interact with trauma survivors [34], nurses working in multiple settings [39, 40] and Italian hospital healthcare workers during the COVID-19 pandemic [41] (see Table 1).

These comparisons indicate that those working in harm reduction are experiencing a pronounced strain on their emotional well-being. Indeed, vulnerability to grief reported in our study approached levels previously observed among bereaved individuals [35].

### Benefits and challenges of providing HR services

Qualitative results indicated that participants found great meaning in their work, which may buffer against some of the stresses of the job. These findings are similar to recent research where HR providers reported that their work gave them a sense of purpose [10, 42]. Our consultation partners supported this; explaining that people do the work out of commitment to their community, not for a salary, and that it is very rewarding.

While levels of burnout and secondary traumatic stress in our study were greater than those previously reported among other healthcare workers (Table 1), we had anticipated that the levels might have been even higher given previous reports [11–13, 43] especially with the added toll of the pandemic [44]. Most respondents had five years or less in the field and it is possible HR providers facing highest levels of burnout and grief had already left the field or were taking time off to cope with stress as has been reported elsewhere [9]. It is also possible that people who were still in practice and experiencing high levels of burnout and vulnerability to grief did not have the emotional capacity nor the free time to respond to this survey. Our consultation partners supported these explanations. Partners confirmed that turnover in HR positions is high, which presents a risk to HR work, as trust decreases if staff changes frequently. Turnover may also occur because people working in HR settings often have casual positions, economic insecurity, and job precarity. These structural vulnerabilities may explain how burnout is experienced [9].

Our study intentionally included a range of HR services provided in a variety of settings, some of which may be more challenging than others. This variability was intentionally included in the study design to reflect the importance of integrated HR across the spectrum of care. However, it may be masking some effects unique to more demanding settings or roles. Partners suggested that the moderate levels of burnout reported may be because it had become normalized among HR providers, which has been previously reported [9]. Partners also indicated that stress comes from every angle (e.g., federal funding reductions, staffing challenges, daily exposure to trauma) and has happened for so long that living with the feelings of burnout was the accepted standard. That being said, partners also explained that the findings likely underreported rates of stress and burnout and suggested there may be a ceiling effect, as reported burnout did not significantly increase with the additional stressors of the pandemic.

The moderate levels of vulnerability to grief were also surprising as they were not in line with previous studies [11]. The scale in our study measured respondents’ ability to cope with grief as opposed to the experience of grief itself. Thus, the moderate levels of grief reported in our study do not indicate that respondents are not surrounded by loss, but rather that they may have found ways to prevent it from weighing too heavily on them. Qualitative responses spoke to the strength individuals gained from their peers. Yet, the interpretation of this finding is likely more nuanced. Our consultation partners indicated that moderate vulnerability to grief was likely a survival mechanism in response to continuous loss. Individuals in this field are saturated with grief, and it feels impossible to add any more grief to what they are already carrying. Partners said it would be overwhelming if they were to stop and try to process the grief, so they “numb out,” develop a “suspension bridge” to avoid the “pit of grief” and keep going.

In a previous examination of HR providers in Canada, participants reported using avoidance to cope with their grief [9]. Our qualitative results and consultation partners reflected that HR providers do not have the luxury of taking time to process their grief. They are motivated to press on and continue their critical work, which has been posed as an example of grief avoidance in the literature [9]. Previous studies that captured the taxing impact of exposure to trauma and experiences of grief used in-person qualitative and ethnographic methods [14, 18, 43]. It is possible that our online survey methodology may not have been sensitive enough to capture such a nuanced experience.

### Services and supports

Respondents were satisfied with the support they received from their coworkers and managers, but also reported a need for more. These contrasting findings may reflect the variability among study participants. Previous literature supported the need for on-site counselling, debriefing, and bonding with colleagues to cope with the challenges of responding to drug toxicity events [13]. Our consultation partners said they also used these activities with some initial success to keep individuals in their roles. They also advised that alternative staffing models were being explored (i.e., taking different roles throughout the week to alter levels of exposure to trauma) to allow individuals time to process their grief. Yet, previous literature highlighted how some HR providers may hesitate to report the emotional impacts of the job to their managers or supervisors, fearing that it may negatively affect their employment [18]. Different interventions should be evaluated to understand the implications for different HR contexts and to scale and spread the most effective strategies.

During the pandemic, strategies that helped alleviate the stress experienced by direct service providers who were deemed essential included monetary compensation, employer recognition, team and peer support, ability to take vacation or leaves of absence, and enhanced access to mental and physical health services [45]. Systematic reviews have shown promising strategies to address stress, burnout, and well-being among healthcare providers. These strategies include cognitive behaviour interventions [46], yoga programs [47–49], mindfulness programs [48, 50, 51], music- and art-based interventions [52] and resilience building programs [46, 53].

Yet, HR providers need access to these supports to be able to benefit from them. Our qualitative results, previous literature [18] and input from consultation partners showed that even when support is available, they are often inadequate. That could be a result of an insufficient number of sessions are covered through benefits, or that services in employee assistance programs do not have the trauma- and grief-informed approach needed to respond to HR provider needs.

Findings also highlight that the healthcare system itself is a barrier to quality care. Respondents indicated that the lack of community services to refer individuals to is a challenge. They reported that their own professional quality of life was affected more by the failures of policies and systems than by their own jobs. These findings indicate a need for system-wide change to better support those who use substances, and, in turn, HR workers as has been previously reported [1, 11, 13, 14, 43]. These changes could address discrepancies between the public and private systems, and regulated and unregulated workers which are seen more often in mental health and substance use health care than other health arenas. Governments could improve access to care, provider well-being, and integration of care by increasing public funding and system capacity for evidence-informed psychotherapy, applying legislation and prescriptive measures to ensure workplace mental health, develop regulations to influence health and disability insurance providers to ensure workplace mental health [54].

### Impacts of the COVID-19 pandemic and the drug toxicity emergency

Generally, respondents reported similar levels of compassion satisfaction, burnout, and self-care in Cycles One and Two. The replication of findings indicates good reliability that the experiences of HR providers were captured accurately. Our consultation partners agreed that the data provides an accurate picture of their experiences, and they were not surprised that the results were similar between the two survey cycles. Partners indicated there were likely no major changes over time because the experiences of providing HR services were already so challenging and rewarding that there was little room for change. Furthermore, they considered themselves an essential service and continued as usual throughout the pandemic. As burnout and compassion satisfaction each relate to the experiences of performing one’s job (which participants indicated they largely continued as normal through the pandemic), these domains may have been less likely to change over time. In contrast, secondary traumatic stress, and vulnerability to grief, which increased in Cycle Two, more so reflect emotional connections between people. These increases are not surprising given the ongoing and escalating drug toxicity emergency, coupled with the additional stress of the pandemic. Respondents reported becoming more sensitive to the well-being of their clients, which has been reported as a reaction to the pandemic elsewhere [55, 56].

Qualitative responses also revealed positive impacts of the COVID-19 pandemic. Some respondents indicated that the pandemic increased the meaning they experienced in their job as they could be a reliable source of support to their clients during a period of turbulence. This was supported by previous findings related to HR provision during COVID-19 [57]. Others reported that the tumultuous time increased their communities’ acceptance of HR services and made them more willing to support others than they had been before the pandemic. Respondents also reported personal benefits such as learning how resilient they could be and their ability to develop new coping skills. These findings suggest that while the pandemic was one of the greatest threats to global well-being, it may also have presented opportunities for post-traumatic growth, where individuals reassess things with positive adaptations following hardships [58]. Initial assessments of frontline nurses in China revealed post-traumatic growth occurring during the pandemic [59, 60]. As we move further into post-pandemic recovery and implementing more solutions to the overdose emergency, mechanisms to foster post-traumatic growth may benefit those working in harm reduction and the community at large.

## Limitations and future research directions

As in all research, there are limitations to the current study that should be acknowledged. As harm reduction can take many forms, there was significant diversity of roles among the study respondents, The experiences of volunteers at a supervised consumptions site could be significantly different from physicians providing opioid agonist treatment. These differences may have masked some of the effects that a certain subset of workers experienced and limits the generalizability of the findings. Moreover, we did not collect data from individuals who had previously provided harm reduction services and then left the practice. It is possible that these individuals may have been those who were the most affected, and their data was not captured in this study.

While our questions focused on the drug toxicity emergency, the experiences reported by respondents cannot be attributed to drug-related harms alone. We did not quantify individuals’ experiences of trauma, nor the degree of trauma they had been exposed to. Additionally, none of the measures used in our study had been validated by HR providers and it is possible that the scales may not have been able to capture nuance of such complex work. For the qualitative analyses, there is always some degree of researcher subjectivity that it incorporated into the analyses, so we acknowledge the potential for unintentional bias.

The nature of the toxicity emergency and political response are constantly evolving, which could have impacted the results. For example, during Cycle One, some data showed decreases in opioid toxicity deaths in Alberta [61]. At the same time, a review of supervised consumptions sites assessed “concerns about [their] impacts on homes, business and communities” [15]. Changes like these may have influenced how individuals replied to the survey’s questions, so their responses might have differed if they had completed the survey earlier or later in the data collection period. To minimize this risk, data were collected over a relatively short period.

Additionally, the distribution of participants from across Canada was not equal. While every effort was made to engage networks from all jurisdictions, a large portion of the sample was from Ontario, Alberta, British Columbia and Manitoba in Cycle One, and from Ontario, Alberta, British Columbia, Manitoba and Quebec in Cycle Two. Similarly, Cycle One had more women than men respond, but Cycle Two had a more equal distribution of gender. It has been documented that women took on a disproportionate amount of child and home care duties during the Covid-19 pandemic [62, 63] and that women were more likely to work in essential roles [64]. It is possible that because of these additional responsibilities, less women had the time available to participate in the second cycle of the study. Finally, the online methodology of the survey may have excluded some individuals providing HR services if they did not have internet access or were not comfortable using online data collection tools. These differences have implications for the generalizability of our findings.

Subsequent analyses could explore how experiences of HR providers differ based on the role, setting or regulatory status. These factors may help provide a more detailed picture of the many nuances identified in the current study. This could inform the development of targeted support or policies that may be most helpful for the diverse HR workforce. Future research could focus on the outcomes observed after implementing trauma-, loss- and gender-informed counselling among HR providers. Respondents indicated that more supports were necessary, but evaluations should be undertaken to ensure benefits and reduce the risk of unintended harms.

As hypothesized in the Discussion section, individuals who are most affected by the emotional toll of providing harm reduction services may no longer be working in this area and were not represented in this survey. Future research should examine the well-being of those who have left the field to better understand long-term impacts of providing services that are both rewarding and challenging and could inform competency, training, and other support initiatives.

## Implications and conclusions

The study’s findings and our partner consultations point to critical strategies to improve the experiences and well-being of those providing harm reduction services.

- A comprehensive healthcare system that integrates harm reduction services more closely with physical, psychological, and social support services would improve access to the services needed by those using substances and those providing harm reduction services.
- Sustainable and reliable federal, provincial, and territorial funding for harm reduction would not only allow the continuity of services but would also remove financial and planning stressors for program directors and staff.
- Specialized gender-, trauma- and grief-informed counselling resources would help prevent further harm to those providing harm reduction services and ensure that the investment in these resources has meaningful outcomes. Employers providing a sufficient number of sessions, amount of financial compensation or both would ensure a benefit is received and sustained.
- Examination and evaluation of equitable staffing models and policies (e.g., mandatory mental health training for leaders, organization-wide mental health policies) would improve the well-being of those providing harm reduction services. Evaluation of these models and policies could lead to the removal of structural vulnerabilities to burnout, such as job precarity and economic insecurity (i.e., leading to adequate pay, benefits, vacation, and sick leave for workers regardless of regulatory status or employment by community or government agencies).
- Bolstering anti-stigma initiatives among the public and healthcare providers in the broader system would increase willingness to seek and offer help, facilitating positive outcomes.

Our study provides an important overview of the challenges and benefits of providing HR services. The challenges outlined by survey respondents must be addressed as a failure to support this essential workforce translates to increased harm among the individuals they serve. The reciprocal nature of the well-being between client and provider has been recommended as a critical factor for improving outcomes in vulnerable populations and their providers. When client and provider well-being is not adequately addressed, it can cascade to take a toll on the healthcare system [45]. Effectively responding to mental health needs may lead to improved outcomes, not only for an individual and the clients they serve but on system capacity and healthcare costs as well. All initiatives to support this workforce should be undertaken with meaningful engagement of people who use substances and HR providers in alignment with the principle “nothing about us without us” [56, 65]. HR services are often among the first place individuals who use substances seek support. Ensuring the well-being of the HR workforce is critical to ensuring quality care is available to people using substances.

## Data Availability

The datasets used in the current study are available from the corresponding author on reasonable request.
